# Stigma, quality of life, and return-to-work outcomes among organ and tissue transplant recipients in Brazil: a cross-sectional study

**DOI:** 10.31744/einstein_journal/2025AO1737

**Published:** 2025-10-30

**Authors:** Edson Arakaki, Érika Bevilaqua Rangel, Janine Schirmer, Bartira Aguiar Roza

**Affiliations:** 1 Universidade Federal de São Paulo Escola Paulista de Enfermagem São Paulo SP Brazil Escola Paulista de Enfermagem, Universidade Federal de São Paulo, São Paulo, SP, Brazil.; 2 Universidade Federal de São Paulo Escola Paulista de Medicina Department of Medicine São Paulo SP Brazil Department of Medicine, Escola Paulista de Medicina, Universidade Federal de São Paulo, São Paulo, SP, Brazil.

**Keywords:** Organ transplantation, Quality of life, Return to work, Social stigma, Surveys and questionnaires

## Abstract

**Objective:**

Organ transplantation improves survival rates and quality of life; however, reintegration into the workforce remains a challenge. Therefore, this study examined the relationship among return-to-work, quality of life, and perceived stigma in Brazilian transplant recipients.

**Methods:**

This cross-sectional study assessed stigma, quality of life (using the Short Form-36), and employment status in 352 transplant recipients. Statistical analyses included Kaplan-Meier survival curves, regression models, and factor analysis to identify key return-to-work and stigma determinants.

**Results:**

The average return-to-work rate was 53.7%, with socioeconomic class, prior employment, and organ transplantation type positively influencing return-to-work. Stigma negatively impacted return-to-work and quality of life, especially Functional Capacity and Mental Health. Older age, racial disparities, and socioeconomic inequities were significant return-to-work barriers.

**Conclusion:**

Return-to-work was observed in 53.7% of transplant recipients, with rates declining over time. Higher return-to-work was associated with pre-transplant employment, higher socioeconomic status, and organ transplant type. Stigma negatively impacted both return-to-work and quality of life. Return-to-work disparities were evident in terms of age, race, and income. Pre-transplant employment has emerged as the strongest return-to-work predictor. Overcoming these structural barriers is essential for optimizing long-term outcomes and ensuring that transplant recipients fully reintegrate into their occupational lives.

## In Brief

Organ transplantation often enhances patients’ physical health and life expectancy; however, professional reintegration remains a challenge. This study highlights how stigma, socioeconomic disparities, and pre-transplant employment history significantly affect the likelihood of returning to work post-transplantation. These findings call for the development of post-transplant rehabilitation strategies and stigma-reduction interventions tailored to support occupational reintegration in this population.

## Highlights

■ The return to work rate was 53.7%, with slower and lower reintegration among low-income recipients.
■ Individuals with higher socioeconomic status (Classes A and B) were over twice as likely to return to work compared with lower socioeconomic status.
■ Higher stigma scores were significantly associated with reduced quality of life in Functional Capacity, Mental Health, and Social Functioning domains (p<0.001).
■ Older age (≥51 years), racial disparities, and unemployment before transplantation were strong barriers to occupational reintegration.
■ Quality of life scores in transplant recipients were consistently lower than those in the general population, with Emotional Role scores reduced by more than 50 points.

## Graphical abstract

**Figure:**
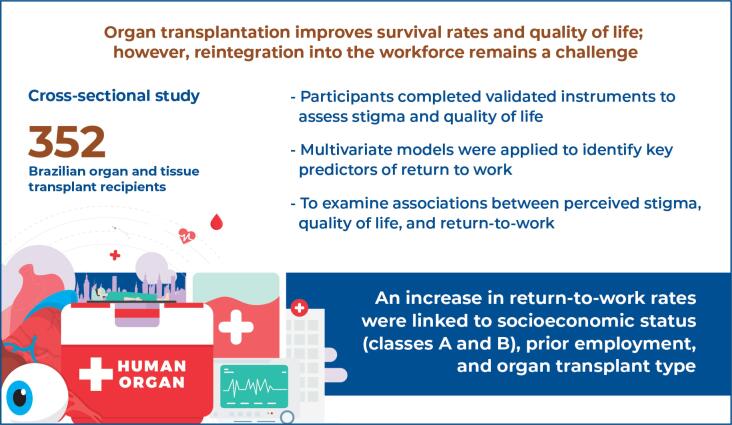

## INTRODUCTION

Organ transplantation has significantly improved the survival rates and quality of life (QoL) in individuals with end-stage organ failure. However, return-to-work (RTW) remains a major challenge for recipients as they face barriers such as stigma, socioeconomic disparities, and the complexities of managing chronic conditions.^(1–4)^

Studies indicate that RTW rates among transplant recipients range from 40% to 80%, but vary depending on professional and social factors.^(5–7)^ White-collar workers and those with lower physical workloads tend to RTW more easily than blue-collar workers.^(8)^ Similarly, patients with rare conditions struggle to RTW due to a lack of awareness among healthcare professionals, patients, and employers regarding their health limitations.^(9)^ Despite these challenges, the disparities in workforce reintegration remain underexplored.

Many transplant recipients who feel physically capable of working still encounter obstacles such as limited job opportunities and concerns about disclosing their health conditions. Stigma plays a key role in these difficulties by affecting social acceptance and professional opportunities.^(10,11)^ Chronic illness-related stigma and visible changes caused by immunosuppressive therapy further reduce workforce participation.^(12,13)^ Stigma is defined as the devaluation of individuals based on illness, disability, or race, which can significantly impact employment prospects.^(14)^

Although transplant recipients often experience improved physical, emotional, and social wellbeing post-surgery, many still struggle with depression, anxiety, and functional limitations.^(3)^ Their overall well-being, particularly in mental health and professional reintegration, remains lower than that of the general population. Individuals with chronic conditions have approximately 30% lower employment rates than their healthy peers.^(15,16)^ However, the specific challenges that influence RTW among transplant recipients remain insufficiently explored.^(15,16)^

## OBJECTIVE

This study examined the relationship among stigma perception, quality of life, and return-to-work outcomes to better understand the barriers faced by transplant recipients and identify strategies to improve their reintegration into the workforce.

## METHODS

**Figure 1:**
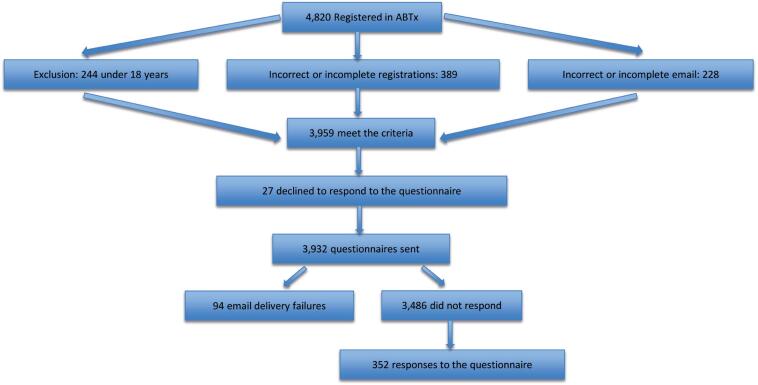
Flow diagram of study patient inclusion

This cross-sectional study surveyed 352 transplant recipients from the Brazilian Transplant Association (ABTx) regarding their demographics, socioeconomic status, QoL (SF36), and stigma. Ethical approval was obtained from the *Universidade Federal de São Paulo* (CAAE: 46796621.4.0000.5505; # 4.793.870) following Resolution 466. Participants aged 18 or older were included only after providing formal consent. Data were collected electronically between July 2 and August 23, 2021, with eligibility requiring at least six months post-transplantation, as shown in figure 1.

Socioeconomic classification was determined using the *Critério Brasil*, established by *Associação Brasileira de Empresas de Pesquisa* (ABEP), which classifies households into five categories (A, B, C, D, and E) based on household assets, education level of the household head, and access to public services. Class A: Wealthiest, with the highest income levels. Class B: Upper- and middle-class with substantial purchasing power. Class C: Lower-middle class with fewer assets and lower income. Classes D-E - Lowest-income groups with limited access to consumer goods and basic services.^(17)^

Chronic illness-related stigma was assessed using a five-item scale developed in Portuguese.^(18)^ This scale was designed to be generic, brief, and applicable to individuals living with any chronic disease or condition requiring continuous treatment or surveillance. Items were selected from existing stigma instruments and refined based on psychometric analyses and theoretical considerations, aiming to reflect the perception of social discomfort, avoidance, and relational difficulties associated with chronic illness.

Participants responded to each item using a 7-point Likert scale ranging from 1 (strongly agree) to 7 (strongly disagree), with higher total scores indicating lower perceived stigma. The five items of the scale were: (1) I feel different from other people because of my condition; (2) Because of my condition, some people feel uncomfortable around me; (3) Because of my condition, I feel that some people avoid me; (4) My condition affects my relationship with friends; and (5) People are afraid of individuals with my condition.

The instrument demonstrated a unidimensional structure and satisfactory psychometric properties, including good internal consistency (Cronbach's alpha=0.82) and evidence of convergent and divergent validity.

### Data analysis

Descriptive statistics: Categorical variables were summarized as frequencies, while numerical variables were summarized as means, quartiles, ranges, and standard deviations. Means were compared using Student's *t*-test or ANOVA, and normality was assessed by the Kolmogorov-Smirnov test. When appropriate, non-parametric tests (Mann-Whitney and Kruskal-Wallis) were applied.

Post-hoc analysis: Duncan's or Dunn-Bonferroni tests were used for multiple comparisons, with a significance level of 5%. Kaplan-Meier analysis was performed to estimate the probability of unemployment persistence post-transplantation, accounting for censored data.

Correlation and regression: Associations between numerical variables were evaluated using Spearman's correlation coefficients. Linear regression models (simple and multiple) assessed the impact of demographic, occupational, and transplant-related factors on stigma and QoL, as measured by the Short Form-36 (SF-36) questionnaire. Non-significant variables were excluded by using the backward elimination method.

Stigma Scale Analysis: Dimensionality was examined using Confirmatory Factor Analysis (CFA) and Exploratory Factor Analysis (EFA), with fit indices including the Root Mean Square Error of Approximation (RMSEA), Comparative Fit Index (CFI), Tucker-Lewis Index (TLI), and normalized chi-square. Internal consistency was assessed using Cronbach's alpha, with values closer to 1 indicating higher reliability. Stigma scores were rescaled to a range of 0 to 100 points.

## RESULTS

The study included 352 participants, with a mean age of 42.4 years (SD=11.3; age range: 18-78 years). Participants were predominantly kidney transplant patients (n=219; 62.2%), followed by liver (n=69; 19.6%), heart (n=16; 4.5%), and bone marrow (n=15; 4.3%) recipients. Other groups included lung (2.8%), combined kidney-pancreas (4.5%), cornea (0.9%), pancreas alone (0.6%), and other transplants (0.6%). Females comprised 56.1% of the sample, 56.5% self-identified as White, and 58.2% were married or in a stable relationship. Approximately 46.6% of the participants belonged to socioeconomic class C, whereas 33.2% belonged to class B. Data were collected from all Brazilian regions (Table 1).

**Table 1:** Sociodemographic characteristics

Category		Results
Gender, n (%)		
	Female	197 (56.1)
	Male	154 (43.9)
Age (years)		
	Mean±SD	341 (42.4±11.3)
	Median (Min - Max)	42.0 (18.0–78.0)
Race, n (%)		
	White	199 (56.5)
	Mixed	108 (30.7)
	Black	33 (9.4)
	Other	12 (3.4)
Marital status, n (%)		
	Married or Stable Union	205 (58.2)
	Single	109 (30.4)
	Separated	26 (7.4)
	Widowed	6 (1.6)
Economic class, n (%)		
	A	27 (7.7)
	B	117 (33.2)
	C	164 (46.6)
	D–E	44 (12.5)
Region, n (%)		
	Central-West	26 (7.7)
	Northeast	64 (18.8)
	North	14 (4.1)
	Southeast	217 (64.1)
	South	52 (14.8)

**Figure 2:**
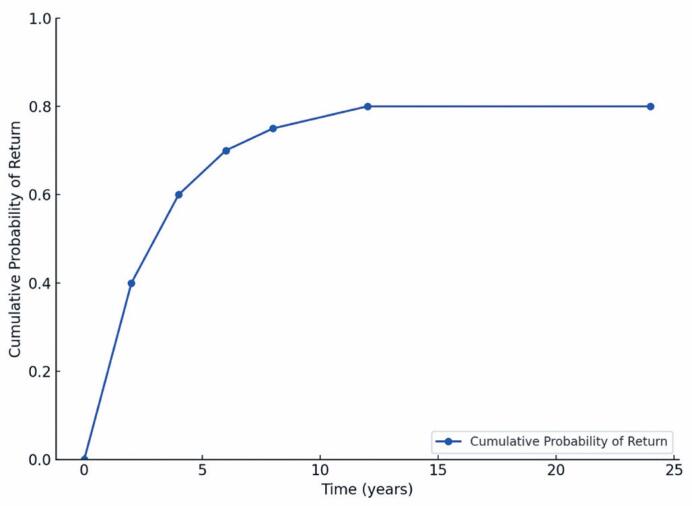
Return to Work Rate (Adapted Kaplan-Meier Curve)

The probabilities of RTW after transplantation were 67.4% within one year, 40.0% within five years, and 33.5% within 10 years (Figure 2). The mean time to RTW was 8.1 years (95%CI=6.7-9.5).

Classes A and B exhibited higher and faster RTW rates, with cumulative curves stabilizing within the first 4-6 years. Conversely, Classes D and E showed significantly lower and slower RTW rates, respectively, than the other groups (Table 2).

**Table 2:** Kaplan-Meier analysis results for return to work

Category		6 Months	1 Year	2 Years	5 Years	10 Years	p value
Economic Class							<0.001
	A	59.26±9.46	51.85±9.27	31.69±9.27	25.35±9.34	12.67±10.11	
	B	68.72±4.32	53.69±4.74	34.75±4.76	22.28±4.56	13.58±4.89	
	C	86.18±2.74	74.69±3.52	62.40±4.07	45.07±4.06	41.51±6.08	
	D-E	90.80±4.39	49.41±5.50	51.02±6.06	76.06±7.06	76.78±7.08	
Donor							0.013
	Deceased	80.84±2.63	68.75±3.16	54.67±3.53	40.67±3.77	28.43±4.04	
	Living	75.14±3.94	61.38±4.81	50.52±4.37	29.18±4.48	16.71±5.69	
Work before transplant							<0.001
	No	96.15±3.77	96.15±3.77	91.78±5.59	81.74±8.38	81.74±8.38	
	Yes	77.74±2.34	69.15±3.77	49.88±2.97	26.23±3.48	22.88±3.78	
Paid activity before transplant							<0.001
	No	92.11±3.79	87.77±4.69	72.38±6.93	25.89±8.80	45.89±8.80	
	Yes	74.66±2.66	60.79±3.03	45.77±3.21	34.43±3.34	24.97±3.40	

The Kaplan-Meier curve represents a decay function, where a decrease in absolute values indicates that individuals have return-towork. In this context, lower numerical values correspond to fewer unemployed individuals.

Kidney recipients of living-donor organs demonstrated higher and faster RTW rates in the early post-transplant years.

In the multivariate model (Table 3), age, race, socioeconomic status, transplant type, and pre-transplant employment status were significantly associated with RTW. Individuals aged ≥51 were 47% less likely to RTW compared with those aged 31-40. Black individuals had a 64% lower likelihood of RTW than white individuals. Socioeconomic status was also a strong predictor. Individuals in classes D-E were 62% less likely to return than those in class C, whereas those in classes A and B were 2.5 and 2.4 times more likely to return, respectively. Bone marrow transplant recipients had a 61% lower RTW rate than kidney transplant recipients. Employment history was a key determinant: individuals unemployed before transplantation were 45% less likely to RTW, and those previously engaged in unpaid work had an 87% lower likelihood.

**Table 3:** Return to work estimate

Category		Adjusted Hazard ratio (HR) (95% CI)	p value
Age range at transplant (ref.: 31 to 40 years)			0.013
	Under 20 years	1.62 (0.85-3.09)	0.141
	21 to 30 years	1.31 (0.90-1.91)	0.157
	41 to 50 years	0.83 (0.53-1.28)	0.392
	51 years and above	0.53 (0.29-0.96)	0.037
Race (ref.: White)			0.031
	Mixed	0.91 (0.65-1.28)	0.596
	Black	0.36 (0.18-0.71)	0.003
	Yellow	0.90 (0.38-2.15)	0.818
Economic Class (ref.: C)			<0.001
	A	2.54 (1.51-4.27)	<0.001
	B	2.44 (1.74-3.41)	<0.001
	D-E	0.38 (0.18-2.15)	<0.001
Transplant type (ref.: kidney)			0.008
	Liver	0.82 (0.56-1.20)	0.304
	Heart	1.34 (0.59-3.05)	0.486
	Kidney and pancreas	1.75 (0.86-3.57)	0.123
	Bone marrow	0.39 (0.18-0.86)	0.019
	Lung	0.55 (0.21-1.46)	0.232
	Others	2.86 (1.20-6.79)	0.017
Paid activity before transplant (ref.: Yes)			<0.001
	No	0.55 (0.34-0.89)	0.016
	Never worked in life	0.13 (0.04-0.39)	<0.001

Only the variables significantly associated with return-towork were considered.

The mean stigma score was 45.5±28.2, ranging from 14.3 to 100.0. Statistically significant differences in mean stigma scores were observed across marital status (*p*=0.003), socioeconomic class (*p*<0.001), and RTW status (*p*<0.001) (Table 4). Separated or widowed individuals reported higher stigma scores than those who were married or in stable unions (mean difference: 14.7 points). Participants in socioeconomic classes A and B had lower stigma scores than those in class C (mean differences: 12.9 and 7.8 points, respectively). Conversely, individuals in classes D-E exhibited higher stigma scores than those in class C (mean difference: 9.6 points).

**Table 4:** Stigma measures and characteristics

Category		N	Mean±SD	p value
Marital status				0.003*
	Married or Stable Union	205	41.7±27.5^A^	
	Single	119	48.8±27.4	
	Separated/Widowed	28	59.4±30.9^B^	
Economic class				<0.001*
	A	27	30.7±20.0^B^	
	B	117	38.3±24.9^B^	
	C	164	48.8±29.0^A^	
	D–E	44	61.4±28.4^A^	
Work post-transplant				<0.001
	No	163	53.3±28.2	
	Yes	189	38.8±26.4	

* The p-value represents the descriptive level of the Mann-Whitney or Kruskal-Wallis test.

Multiple pairwise comparisons using the Dunn-Bonferroni method indicated that groups (A) and (B) have significantly different means.

Furthermore, individuals who RTW or started working after transplantation had lower stigma scores (mean difference: 9.8 points) than those who remained unemployed.

**Figure 3:**
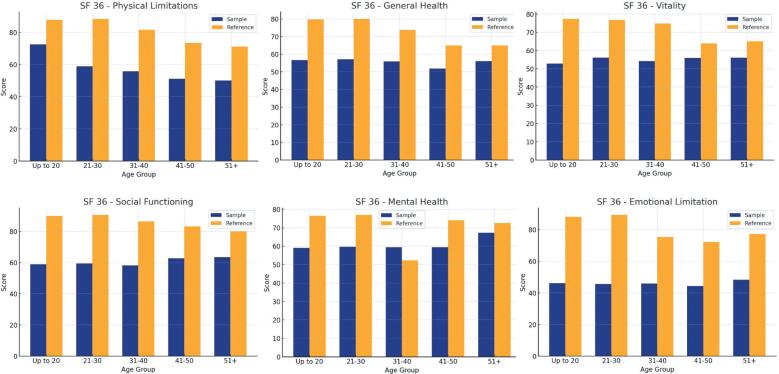
SF-36 statistic summary for the general population vs. study population

Analysis of the SF-36 dimensions between the reference general population and the study sample consistently revealed lower scores across all age groups and dimensions, indicating poorer physical and mental health in the study population than in the general population (Figure 3).

An increase of one point in the stigma perception score was significantly associated with a decline across multiple dimensions of QoL, as assessed by the SF-36. Significant reductions in Functional Capacity (0.24 points), General Health (0.20 points), Vitality (0.25 points), Social Functioning (0.37 points), and Mental Health (0.33 points). Additionally, a 1-point increase in stigma perception was linked to worsening Physical Role Limitation (0.40 points) and Pain (0.23 points) (Figure 3).

## DISCUSSION

Our findings highlight the challenges associated with post-transplantation RTW. The probabilities of RTW were 67.4%, 40.0%, and 33.5% at 1, 5, and 10 years post-transplantation, respectively, with an average RTW rate of 53.7%. Multivariate analysis identified age, race, socioeconomic status, transplant type, and pre-transplant employment status as independent predictors of RTW. Stigma and QoL were also relevant dimensions, with the results indicating their association with RTW outcomes and their potential influence on post-transplant reintegration.

The study analyzed a sample of 352 individuals registered at ABTx, with a broad geographic representation from all Brazilian states. The state of São Paulo accounted for 41.2% of respondents, indicating a significant concentration in this region. Other states with higher representation included Rio de Janeiro (10.2%), Minas Gerais (8.8%), and Paraná (6.8%), whereas several northern and northeastern states participated minimally. This uneven distribution mirrors national disparities in organ transplantation infrastructure, as regions with more developed healthcare systems, particularly in the Southeast and South, perform proportionally more transplant procedures.^(19)^

The findings indicate that RTW is most prevalent within the first year following transplantation, aligning with evidence from the Swiss Transplant Cohort Study, which reported an employment rate of 49.8% at 12 months post-procedure.^(20)^ After 10 years, socioeconomic factors and social support emerged as the most significant determinants of RTW.

The organ donor type is associated with fewer complications and a better QoL for recipients.^(21,22)^ Consistent with previous studies,^(23,24)^ donor type also influenced RTW rates, with higher employment rates among living-donor recipients. However, this finding was not significant in multivariate analysis. Pre-transplant employment status was a strong predictor of RTW success, with previously employed individuals demonstrating an 80% cumulative probability of RTW compared with less than 30% among those unemployed before transplantation.

Return-towork rates among transplant recipients vary widely, ranging from 40% to 80%, and are influenced by the type of transplant and the availability of supportive resources. In our sample, the overall RTW rate was 53.7%, consistent with international rates. Our analysis showed that bone marrow transplant recipients were 61% less likely to achieve RTW compared with kidney transplant recipients, underscoring the significant disparities in post-transplant occupational reintegration. This aligns with the existing literature, which reports that nearly 40% of young adult hematopoietic cell transplantation (HCT) survivors are out of work three years post-HCT.^(25)^ Common obstacles include chronic graft-versus-host disease (GVHD), prolonged immunosuppressive therapy, and decreased physical functioning, all of which impair work readiness and sustainability.^(25,26)^ Conversely, higher RTW rates among kidney transplant recipients (typically between 40% and 65%) have been attributed to fewer postoperative complications and more favorable recovery trajectories.^(27)^

The SF-36 scores for transplant recipients were consistently lower than those of the general Brazilian population across all dimensions, except Mental Health, in the 35-44 age group. The largest disparity was observed in the Emotional Role, with a reduction of over 50 points compared with the general population, emphasizing the need to address emotional and social factors during post-transplant rehabilitation. Although QoL improved after transplantation, the scores remained lower than those in the general population.^(28)^

Our study identified a strong correlation between socioeconomic status, QoL, stigma, and RTW. Individuals in socioeconomic classes A and B scored significantly higher across all SF-36 dimensions than those in classes D and E, and reported lower stigma levels than those in classes C, D, and E. Additionally, individuals in classes C, D, and E had a 40% lower likelihood of RTW than those in classes A and B. Consistent with the literature, these findings highlight the critical role of socioeconomic factors, including educational level and type of occupation, in determining RTW.^(29,30)^

Lung transplant recipients experience substantial improvements in physical capacity, daily functioning, and social engagement, particularly within the initial months post-transplantation.^(31)^ However, emotional well-being and vitality remained suboptimal.^(32)^ The interaction between physical and mental health is particularly pronounced as chronic pain and metabolic complications from immunosuppressive therapies further exacerbate these difficulties.^(31)^

Additionally, marital status, age, and donor type influenced RTW. Married individuals reported higher QoL scores, particularly for Functional Capacity and Vitality, aligning with evidence linking social support to better outcomes in chronic diseases.^(30)^ Individuals over 51 years were less likely to RTW, reflecting the labor market challenges faced by older individuals.^(31,32)^

Historically, stigma has shaped societal responses to diseases, from leprosy and tuberculosis to HIV/AIDS,^(33–35)^ often marginalizing affected individuals. The negative effects of stigma on QoL and RTW are consistent with those of international studies, showing that stigma related to chronic illnesses reduces confidence, self-esteem, and social integration.^(36)^ Although the instrument was originally designed for the general population living with chronic conditions,^(18)^ its concise structure and generic wording allowed its effective use among solid organ and bone marrow transplant recipients. In the present study, higher stigma scores were significantly associated with lower QoL scores across all SF-36 domains, with the greatest impact observed in Functional Capacity and Mental Health. These findings underscore the urgent need for targeted stigma-reduction strategies, such as public awareness campaigns and workplace accommodations, which can be adapted to diverse cultural and economic contexts.

Our findings support the hypothesis that organic and functional limitations caused by transplantation heighten stigma perception, which, in turn, exacerbates these limitations and reduces the likelihood of RTW.^(37,38)^ Expanding the concept of stigma to include less visible conditions such as organ transplantation is essential to mitigating its substantial negative impact on patients’ psychosocial well-being, as reflected in SF-36 scores. Stigma can lead to shame, self-limitation, and discrimination, which directly impair both QoL and RTW.^(39)^

### Limitations

Our study had a few limitations. The cross-sectional design limits its ability to infer causality, as it captures data at a single point in time without establishing temporal relationships between variables. The sample largely comprised participants from the southeastern region of Brazil, particularly São Paulo, where most transplant centers are located. This regional concentration may limit the generalizability of the findings to other areas of the country, such as the North and Northeast, which face significant disparities in healthcare infrastructure, access to transplantation, and socioeconomic conditions. The absence of clinical data on comorbidities, such as cardiovascular disease, metabolic syndrome, and mental health disorders, is also a limitation, as these conditions are common among transplant recipients and can negatively affect both quality of life and return-towork outcomes. Although psychometric analyses have been conducted, the stigma scale has not been specifically validated for transplant populations, thereby limiting interpretive stigma-related findings.

## CONCLUSION

The findings indicate that post-transplantation return-to-work tends to decrease over time. More sustained rates were observed among individuals with higher socioeconomic status, whereas lower-income groups had slower and lower return-to-work rates. Increased return-to-work rate was associated with socioeconomic status (classes A and B), prior employment, and organ transplant type. Stigma negatively impacted return-to-work and quality of life, particularly in Functional Capacity and Mental Health. Older age, racial disparities, and socioeconomic inequities were also significant barriers to return-to-work.

Addressing barriers such as stigma, employer biases, and the lack of post-transplant rehabilitation programs can enable healthcare systems to help transplant recipients not only survive and thrive, allowing them to make meaningful contributions to society and the economy.
